# Advances in intelligent diagnosis methods for pulmonary ground-glass opacity nodules

**DOI:** 10.1186/s12938-018-0435-2

**Published:** 2018-02-07

**Authors:** Jing Yang, Hailin Wang, Chen Geng, Yakang Dai, Jiansong Ji

**Affiliations:** 1 School of Biomedical Engineering, University of Science and Technology of China, Hefei, 230026 People’s Republic of China; 2 Suzhou Institute of Biomedical Engineering and Technology, Chinese Academy of Sciences, Suzhou, 215163 People’s Republic of China; 3 Radiology Department and Interventional Radiology Center, The Fifth Affiliated Hospital of Wenzhou Medical University, Affiliated Lishui Hospital of Zhejiang University, The Central Hospital of Zhejiang Lishui, Lishui, 323000 People’s Republic of China

**Keywords:** Ground glass nodules, Lung cancer, Early diagnosis, Nodular signs, Prediction models, Intelligent diagnosis

## Abstract

Pulmonary nodule is one of the important lesions of lung cancer, mainly divided into two categories of solid nodules and ground glass nodules. The improvement of diagnosis of lung cancer has significant clinical significance, which could be realized by machine learning techniques. At present, there have been a lot of researches focusing on solid nodules. But the research on ground glass nodules started late, and lacked research results. This paper summarizes the research progress of the method of intelligent diagnosis for pulmonary nodules since 2014. It is described in details from four aspects: nodular signs, data analysis methods, prediction models and system evaluation. This paper aims to provide the research material for researchers of the clinical diagnosis and intelligent analysis of lung cancer, and further improve the precision of pulmonary ground glass nodule diagnosis.

## Background

Lung cancer is one of the major causes of death in cancer patients, accounting for about 27% of all cancer deaths [1]. Pulmonary nodules are one of the early symptoms of lung cancer, and the correct diagnosis results can effectively improve the survival rate of patients with early lung cancer. According to the content of solid ingredients, pulmonary nodules can be divided into solid nodules and ground glass nodules (GGNs). At present, GGNs are more likely to be malignant than solid nodules [2]. But the research on GGNs is not enough. Therefore, this article mainly introduces the new progress of the diagnosis in the benign and malignant pulmonary ground glass nodules.

**Fig. 1:**
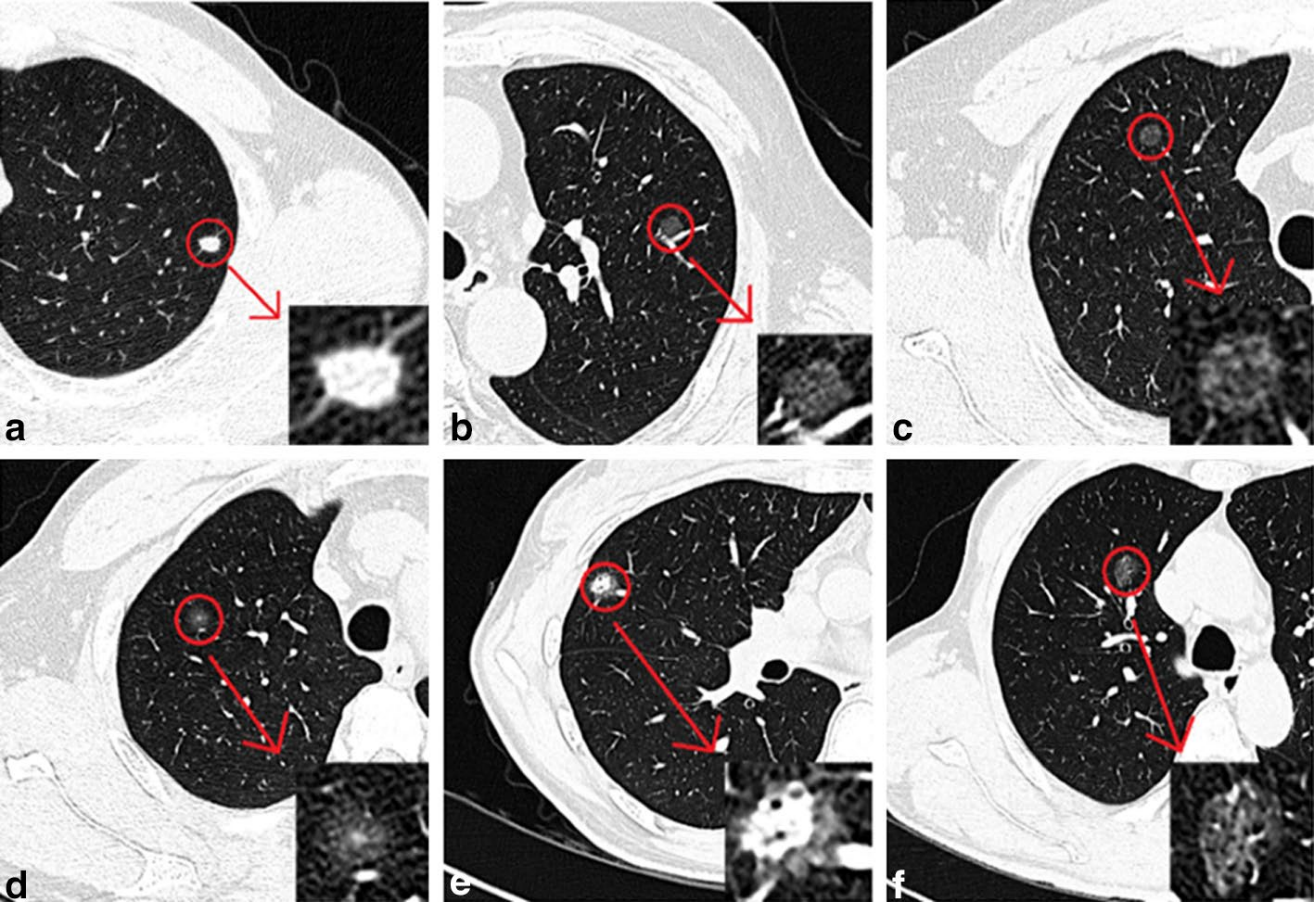
Some different pulmonary nodules. **a** Irregular solid nodules in left lower lobe. **b** Pure ground glass nodules in left upper lobe. **c** Pure ground glass nodules in right upper lobe. **d** Mixed ground glass nodules in right upper lobe. **e** Mixed ground glass nodules in right middle lobe. **f** Mixed ground glass nodules in right middle lobe

Pulmonary ground glass nodules are often referred to as pulmonary ground glass opacity (GGO). It is defined as the increased attention of the lung parenchyma without obscuration of the pulmonary vascular markings on CT images [3]. In addition, it is also known as the subsolid nodule (SSN), which is a characteristic but non-specific imaging sign. According to the presence of solid ingredients in the nodules, the GGO is divided into two types: the mixed ground glass opacity (mGGO) and the pure ground glass opacity (pGGO) [4]. The mGGO is also known as part-solid GGN, which contains solid part and non-solid part. But pGGO does not contain solid ingredients. The classification of pulmonary nodules is shown in Table 1. Here are some clinical images to describe the classification of pulmonary nodules in Fig. 1.

**Table 1:** The classification of pulmonary nodules

The type of nodule	The name of the subclass	Solid part	Non-solid part
Solid nodules		+	−
Ground glass nodules	Pure ground glass opacity	−	+
	Mixed ground glass opacity	+	+

“+” means containing this ingredient. “−” means that this component is not included

At present, there are already common processes for the lung nodules detection, including image acquisition, pre-processing, pulmonary parenchyma segmentation, nodule detection, and false positive reduction [5]. This process is consistent with the flow of the GGNs detection in the computer-aided detection (CAD) system. In the study of the detection and diagnosis of GGNs, the difficulty is the difference of the main emphasis of the researchers, the properties of datasets, the complexity of the system and the performance of evaluation methods. Considering the diversity of the existing works, this paper presents a review on GGNs diagnosis since 2014 to provide the research material for researchers of the clinical diagnosis and intelligent analysis of lung cancer. It can be divided into four sections including nodular signs, data analysis methods, prediction models and system evaluation. In the first section, the common nodular signs are enumerated. Subsequently, data analysis methods are used to find predictors among the nodular signs to predict benign and malignant GGNs, which can provide a reliable basis for the establishment of the follow-up benign and malignant prediction models. Next, establish predictive model for the differential diagnosis of benign and malignant nodules. The subsystem architecture for the diagnosis about GGNs in the CAD system can then be developed, hence summarize system evaluation methods to evaluate these system performances in the final section. Each section will be explained in further detail below.

## Nodular signs

Objective and accurate understanding of nodular signs has important significance for benign and malignant diagnosis of GGNs. Combining the current research progress, some common nodular signs of pulmonary ground glass nodules in CAD system are described below. These nodular signs are respectively: nodule size or lesion diameter, lobulation and spiculation, smoking history, lung cancer history, the proportion of solid components within the lesion, pleural indentation, air bronchogram, and vascular convergence sign.

### Nodule size or lesion diameter

Cho et al. [6] concluded that larger nodules were independent predictors of malignant tumors by analyzing 356 cases of ground glass nodules from 324 patients in January 2009–October 2013. Ueda et al. [7] found that the changes of CT images were associated with pathological findings. All non-malignant lesions were non-enlarged tumors, and all increased tumors were diagnosed with adenocarcinoma. Studies of Xu et al. [8] have shown that older patients with a greater nodular diameter are more likely to suffer lung cancer, and more than 10 mm of subsolid nodules are statistically significantly associated with malignant tumors. It can be seen that the nodule size or lesion diameter has a very close relationship with the benign and the malignant degree of pulmonary ground glass nodules. The greater the nodules indicate, the more likely the nodule is to be malignant.

### Lobulation and spiculation

When a portion of the pulmonary nodule surface is wavy or fan-shaped, the edge of the nodule is described as the lobulation. Similarly, when the edge of the pulmonary nodule extends to the lung parenchyma, the nodule is called the spiculation. Hu et al. [9] conducted a study to determine the imaging characteristics that contributed to the differential diagnosis of solitary GGNs. Through the research on pathological examination results of 112 cases of 112 patients with solitary GGNs after surgical resection, it’s found that benign and malignant nodules have significant differences in lobulation, spiculation and other aspects. Liu et al. [10] presented a systematic approach to analyze low resolution CT images of 172 patients. Excluding nodule size in dataset 2, four best features to predict the malignant indicators of the original nodules were found, including lobulation and spiculation. Zhao et al. [11] focused on spiculation and found that the pulmonary resolving nodules and malignant nodules have similar CT features. So the lobulation and spiculation are the important indicators of malignant tumors.

### Smoking history

The use of low-dose CT lung cancer screening can reduce lung cancer mortality in high-risk smokers [12]. However, it is far from enough. Some researchers have begun to study the relation between smokers and lung cancer mortality [13, 14]. She et al. [13] identified preinvasive lesions and invasive pulmonary adenocarcinomas (IPAs) based on data from patients with solitary pulmonary pure GGNs that had been confirmed from January 2009 to September 2015. It was found that smoking status was one of the predictors of the invasive extent. The longer the smoking time and the greater the amount of smoking, the higher the likelihood of suffering from malignant tumors.

### Lung cancer history

Tamura et al. [15] reviewed 63 cases of pure ground glass nodules to assess the relationship between clinical and imaging findings and pulmonary GGN progression, identifying risk factors that predict pGGO lesions. In the growth group, pGGO lesions were closely related to the high mean computed tomography (m-CT) values and lung cancer history, but not close to smoking habits and GGN shape. It can be seen that lung cancer history is one of the independent predictors of future changes in GGN lesions. In the histological subtypes, the effects of clustering on lung cancer families still exist after a variety of confounding factors such as socioeconomic status and smoking habits. Wille et al. [16] demonstrated that the age and history of lung cancer in the Danish Lung Cancer Screening Trial (DLCST) have a significant predictive effect on the lung cancer risk of solitary pulmonary nodules (SPNs).

### The proportion of solid components within the lesion

The pulmonary nodules in the order of the malignant risk are: mGGO>pGGO>solid nodules. In [2] and [14], it demonstrated the GGN is more likely to be malignant than solid nodules. But the proportion of solid components within the lesion is also important. Choi et al. [17] concluded that the presence of part-solid nodules were significantly associated with malignant lesions. Among them, the proportion of solid ingredients in GGNs and lung cancer clinicopathological staging is associated. The solid component of advanced lung cancer is significantly more than early lung cancer [18]. In addition, the focal ground glass opacity (fGGO) is an important sign of lung cancer. Its incidence of stage I lung cancer is higher than benign lesions. Malignant rates of mGGO, pGGO and solid lesions were 75.0, 60.0 and 48.2%, respectively. Studies showed that the later stage of lung cancer, the lower the proportion of pGGO, and the higher the proportion of mGGO. If fGGO lesions contain solid ingredients, it is the highest possibility of malignant lesions. Followed by pGGO, the possibility of malignant lesions is relatively low [19].

### Pleural indentation

Pleural indentation, it is the linear, curly or star-shaped shadow between the tumor and pleura, which is closely related to the benign and malignant diagnosis of GGNs. In [10], researchers predict the malignant degree of the pulmonary nodules by using the imaging features. The pleural indentation is one of the four best feature sets in the dataset 1 that contains the size measurement. In order to study the pathological findings of pulmonary pGGO lesions and to assess the likelihood of invasive malignancies, Ichinose et al. [20] analyzed 191 patients with GGO lesions (114 cases of pGGO and 77 cases of mGGO) from January 2008 to December 2010 in 160 patients. They found that invasive lung cancer accounted for 12% of pGGO lesions, most of which showed pleural indentation. In [13], univariate analysis or multivariate analysis showed that pleural indentation was a predictor of lung adenocarcinoma invasion.

### Air bronchogram

Air bronchogram, refers to the lesion that we can see the translucent bronchial shadow in the lung tissue area. Dai et al. [21] analyzed the pure GGNs data from 71 patients with primary tumors from June 2010 to December 2013. It was found that air bronchogram is one of the potential factors of pure GGNs. And it helps to determine the benign and malignant synchronous pure GGNs before surgery. In analyzing the CT characteristics and pathologic classification of patients with pGGO in early lung adenocarcinoma, Jin et al. [22] concluded that there was a close relationship between air bronchogram and histological invasiveness. It can help to predict the invasive degree of pGGO in early lung adenocarcinoma.

### Vascular convergence sign

In [9], Hu et al. confirmed that vascular convergence sign is an important indicator of malignant lesions according to pathological examinations of 112 patients with 112 cases of solitary GGNs after surgical resection. It is one of the risk factors for characterizing malignant tumors. In the literature [14], the author also said that in addition to lobulation and spiculation, the vascular convergence sign is also one of the signs of malignant pulmonary solid nodules in imageology.

Yip et al. [23] reviewed and reanalyzed the existing literature, and concluded that lung cancer characterization for GGNs was a slow process. With the progress of CT scanning, GGNs are more frequently detected in chest CT scans of lung cancer and other indications, suggesting that physicians can have more time to cure patients with non-solid nodules. Although CT-guided percutaneous needle aspiration biopsy is an effective way to diagnose subsolid nodules in pathologic diagnosis [24], with the rapid development of computer technology, imaging signs to assess nodular growth and the benign and malignant situation are very useful in recent years. Especially in further image analysis, the use of CT enhancement studies and positron emission tomography (PET) techniques are useful in identifying malignant latent factors of GGNs [25]. The list of nodular signs associated with the benign and malignant identification in the relevant literature is shown in Table 2. It’s easy to see that the most frequently used nodular signs are nodule size or lesion diameter, lobulation and speculation, smoking history, pleural indentation, and lung cancer history. The rational use of several nodular signs can have a good diagnostic value for early lung cancer.

**Table 2:** The list of nodular signs in the relevant literature

Nodular signs	Literature
Nodule size or lesion diameter	[6–10, 12–18, 21, 22]
Lobulation and spiculation	[9–11, 13–19, 21, 22]
Smoking history	[7, 12–15, 17, 21]
Pleural indentation	[10, 13, 17–20]
Lung cancer history	[12, 14–17]
The proportion of solid components within the lesion	[9, 15, 18, 19]
Air bronchogram	[18, 21, 22]
Vascular convergence sign	[9, 14, 19]

## Data analysis methods

Based on the previous study of nodular signs, many scholars use statistical methods to assess the importance of these nodular signs and find predictors to predict benign and malignant GGNs effectively. Meanwhile, using data statistical methods can provide a reliable basis for researching the follow-up benign and malignant prediction models and differentiating malignant from benign pulmonary nodules. In addition to basic comparative statistics including Student’s t test, Chi square test, Fisher’s exact test, there are relatively novel or special data analysis methods such as density histogram, nomogram, volume doubling time (VDT) and mass doubling time (MDT). The appropriate use of data statistical methods can help radiologists to determine and identify the benign and malignant pulmonary nodules and help to reduce the pressure of radiologists.

Kamiya et al. [26] used the kurtosis and skewness of the density histogram to evaluate the characteristics of the pulmonary nodules. They found that the method was effective in assessing the characteristics of nodules and predicting the benign and malignant nodules. By analyzing 93 cases of pulmonary nodules (72 cases of malignant nodules and 21 cases of benign nodules), they found that the peak of malignant lesions was greater than benign nodules. It can be seen that the kurtosis and skewness of the density histogram can help distinguish benign and malignant nodules. In [13], the authors established and validated a new nomogram that identifies IPAs from preinvasive lesions in solitary pure GGN patients. It was found that lesion size, lesion edge, lesion shape, mean CT value, pleural indentation and smoking status were significantly correlated with the degree of invasion in univariate analysis or multivariate analysis.

Song et al. [27] estimated the volume doubling time and mass doubling time of low-dose CT persistent pulmonary subsolid nodules in patients with no history of malignant tumors, 97 SSNs of 97 patients were divided into three groups: Group A, pure GGNs; Group B, part-solid GGNs, the solid ingredients ≤ 5 mm; Group C, part-solid GGNs, the solid ingredients > 5 mm. The VDTs and MDTs of Group A and Group B were significantly higher than those in Group C. In other words, the VDTs and MDTs of pure GGNs and part-solid GGNs with solid ingredients ≤ 5 mm were significantly higher than those in part-solid GGNs with solid ingredients > 5 mm. Scholten et al. [28] also calculated the mass doubling time of pulmonary nodules by measuring the maximum diameter, volume, and mass. He analyzed 264 pulmonary subsolid nodules of 234 participants. The results showed that although persistent SSNs according to pathologic analysis had a higher rate of malignancy, it rarely developed into clinically malignant lesions.

Das et al. [29] analyzed the data from 32 patients with 35 pulmonary nodules (excluding pGGO, containing only solid nodules and mGGO), assessed the feasibility of diffuse kurtosis imaging (DKI) in the human lungs and compared its diagnostic value with standard diffusion weighted imaging in the differential diagnosis of benign and malignant pulmonary nodules. The results showed that human pulmonary DKI is clinically feasible. In theory, the mean peak (MK) of DKI in malignant lesions is significantly higher than that in benign lesions, showing a higher non-Gaussian diffusion. Dhara et al. [30] used differential geometry-based techniques to calculate the spiculation, lobulation, and sphericity using the binary mask of the segmented nodules. They proved that the boundary roughness of pulmonary nodules is an important sign of its degree of malignancy.

In addition, there are some studies on data analysis methods which can also contribute to the prediction of benign and malignant pulmonary nodules. Dhara et al. [31] studied the use of support vector machines (SVM) in the benign and malignant identification of pulmonary nodules (including solid nodules, subsolid nodules and non-solid nodules) using shape-based, edge-based, texture-based features to characterize these pulmonary nodules, with 891 nodules to verify the proposed classification program is superior to competing with other technologies. Kaya et al. [32] also explored the contribution of nodular features in the prediction of malignant lesions and proposed a classification method based on a weighted rule method to predict the benign and malignant pulmonary nodules. The experimental results showed that the classification results of malignant prediction using nodular features can be improved. In the literature cited above, data analysis methods and related nodular signs are shown in Table 3.

**Table 3:** The list of the data analysis methods in the relevant literature

Nodular signs	Data analysis	Literature
Nodule size or lesion diameter, lobulation and spiculation	Density histogram kurtosis and skewness	[26]
Nodule size or lesion diameter	Volume doubling time, mass doubling time	[27]
Nodule size or lesion diameter	Mass doubling time, Student’s t test	[28]
Nodule size or lesion diameter	Diffusion kurtosis imaging	[29]
Lobulation and spiculation	Differential geometry-based	[30]
Nodule size or lesion diameter, lobulation and spiculation	Support vector machine	[31]
Lobulation and spiculation	Weighted rule based	[32]

Some common nodular signs, including margin, volume, mass, lobulation, sphericity and texture, can be seen from Table 3. It can obviously see that scholars use different data statistical methods due to the different selection and combination of nodular signs. One of limitations is the inability to compare these data analysis methods. Although specific matters need to analyse specifically, finding the most widely used and recommended data analysis method is a problem needs to be solved.

## Prediction models

In order to identify the malignant lesions in GGNs better, some scholars, with the basis of nodular signs and data statistical methods, established the prediction models to assess the patient’s malignant rate. The results of the research on the benign and malignant prediction models of GGNs are summarized as follows.

Shinohara et al. [33] collected age, smoking status, lung cancer history, nodule size, location, and spiculation data from 241 patients using the Mayo [34] prediction model, which was based on the American College of Chest Physicians (ACCP), to identify the probability of malignancy (POM) for each patient. Significant differences were found between benign and malignant patients in terms of age, smoking history, nodule size, and spiculation. But another finding was that the predictive model proposed by the ACCP guideline was not satisfactory in the differential diagnosis of benign and malignant solitary pulmonary nodules.

Zheng et al. [35] proposed a modified model for preoperatively predicting malignancy of the solitary pulmonary nodules, as shown in Table 4. Two-thirds of the 846 patients from the Fujian Medical University Union Hospital were randomly selected as a derivation set, and the remaining one-third was used as a validation set. They divided lesions according to the proportion of GGO. The probability of malignancy is e^x^/(1 + e^x^), where e is the base of natural logarithms. This prediction model accurately identified the malignant lesions of solitary pulmonary nodules, especially with 50% or greater of the GGO, which is superior to the Mayo Clinic model.

**Table 4:** The list of a modified preoperatively prediction model

GGO (%)	Formula
< 50	x = − 7.442 + (0.051 × age [years]) + (0.711 × presence of symptoms) + (0.066 × serum total protein [g/L] concentration) + (0.032 × diameter [mm]) + (1.071 × lobulation) + (1.220 × calcified nodes)
≥ 50	x = − 6.192 + (− 0.924 × sex) + (0.042 × FEV1%) + (0.131 × diameter [mm]) + (2.424 × calcified node [s])

In [16], Wille et al. evaluated the discriminant properties of the PanCan model by conducting the Danish Lung Cancer Screening Trial (DLCST) in 1152 pulmonary nodules of 718 patients. It was found that the PanCan risk prediction model [36] has a high risk of lung cancer identification with solitary pulmonary nodules. Risk prediction of lung cancer is mainly based on the size of pulmonary nodules. In addition, spiculation, age, and family history also have a significant predictive effect. Van Riel et al. [37] compared the performance of the PanCan model, Lung-RADS [38] and 1.2016 NCCN guidelines [39] when identifying benign and malignant pulmonary nodules and determined the effect of different diameter measurement methods on model performance. The results showed that the performance of the PanCan model in the differential diagnosis of benign and malignant pulmonary nodules was superior to Lung-RADS and 1.2016 NCCN guidelines. The 1.2016 NCCN guidelines were most sensitive to the definition of nodule size. But the definition of nodule size doesn’t significantly affect Lung-RADS and the PanCan model. Takahashi et al. [40] proposed a new classification criterion of lung adenocarcinoma according to the theory from The International Association for the Study of Lung Cancer, American Thoracic Society, and European Respiratory Society (IASLC/ATS/ERS). The new classification criterion was based on histology, clinic, molecular, radiologic, surgical and other multidisciplinary methods. They found tumor invasiveness of lung adenocarcinoma is an important prognostic factor of pathologic stage IA lung adenocarcinoma. And the GGO ratio (the GGO ratio (%) defined as follows: (1 − [maximum dimension of consolidation on the lung window setting/maximum dimension of the tumor on the lung window setting]) × 100) is one of the effective indicators of invasive lung adenocarcinoma.

Recently, many researchers have also applied end-to-end learning machines in medical image analysis field. Two kinds of end-to-end machine learning methods are massive-training artificial neural networks (MTANNs) and convolutional neural networks (CNNs). Shen et al. [41] used the CNNs to establish an end-to-end computing architecture and studied high-level suspiciousness specific features for lung nodule classification with the Multi-crop Convolutional Neural Network (MC-CNN), which was robust in the feature extraction and malignancy suspiciousness classification of pulmonary nodules. Due to the removal of nodule segmentation and hand-crafted feature (e.g., texture and shape compactness) engineering work, the proposed method can simplify conventional lung nodule malignancy suspiciousness classification, which can help researchers to assess the uncertainty of malignancy. Tajbakhsh et al. [42] compared two end-to-end machine learning methods in the detection of pulmonary nodules and the performance of benign and malignant pulmonary nodules. Experiments showed that the performance of MTANNs in the detection and identification of pulmonary nodules was higher than that of CNNs when using only limited training data. When using a larger training dataset, the performance gap became less evident even though the margin was still significant.

The prediction models with a better performance have a significant predictive effect on the lung cancer risk. They can help radiologists reduce the reading time and improve the diagnostic accuracy. The traditional prediction models like the PanCan model have a better performance in identifying benign and malignant pulmonary nodules, but not yet enough. Besides, these prediction models that have been mentioned consider GGNs, not aimed at them. Lack of pertinences is one of the bottlenecks.

## System evaluation

Combined with the first three stages, the complete subsystem architecture for the diagnosis about GGNs in the CAD system can be designed or built. The researcher adopted different evaluation methods to obtain and identify the suitable subsystem according to the individual specific conditions. The most commonly used evaluation methods are the receiver operating characteristics (ROC) and area under the receiver operator characteristic curve (AUC), such as [10, 22, 26], and so on.

Both [9] and [43] use binary logistic regression analysis methods to compare the relationship between imaging features and histopathological classification of benign and malignant pulmonary nodules.

Cha et al. [44] said that there was no single effective method for differential diagnosis of pulmonary nodules. But the growth rate measurement using volumetry, evaluation of tumor vascularity on dynamic helical CT, dual-energy CT and MRI, and physiologic evaluation of PET/CT can be used to characterize nodules. Shin et al. [45] also used volumetry. They measured interval changes in nodule volume using CT nodule volumetry software. The study showed that in low-dose CT scans if solid subcentimeter nodules were stable during the initial 2-year follow-up period, they can be regarded as benign lesions. Subcentimeter GGNs are more likely to grow than solid nodules, so they require a longer follow-up period.

Kobayashi et al. [46] used Cox proportional hazards and logistic regression models for evaluation. Experiments showed that the smoking history and initial lesion diameter were closely related to the growth of GGNs. Yanagawa et al. [47] also used the automated computer program to analyze the volume of lung adenocarcinoma in stage I, with the Cox proportional hazards and logistic regression model analysis. Two volume measurement methods (solid volume, ≥ 1.5 cm^3^; solid volume ratios, ≥ 63%) were found as independent predictors which was associated with an increased possibility of recurrence and/or death in patients with stage I adenocarcinoma.

There are a number of other evaluation methods. For example, Zhao et al. used means of generalized estimating equation analysis to compare CT features of resolving nodules with nonresolving nodules (stable and malignant) [11]. Gomez Saez et al. [48] used the Poisson regression method to calculate the risk and mortality of lung cancer in patients with solitary pulmonary nodules and found that nodule size, spiculation, and other imaging features were associated with lung cancer. Han et al. [49] quantitatively compared the characteristics of pulmonary nodules in CT images to reflect the powerful characteristics of malignant tumors. Unsworth [50] used three-dimensional morphological changes and edge sharpness analysis in the three-dimensional CT scan of malignant pulmonary nodules to complete false-positive reduction and malignancy classification. Jiang et al. [51] proposed a modified inflammation-based score and validated its effect on the malignant prediction of fGGO in the lungs.

Some major evaluation methods in all references are shown in Table 5. It is easy to see that the most commonly used evaluation methods are ROC, AUC, univariate analysis or multivariate analysis. These evaluation methods have stronger applicability and generality. Other methods use less due to the particularity of the application. It should not be supposed that the most used method is always better. It still depends on specific cases. In addition, none of research methods is limited to one evaluation method such as [16] and [22]. Everyone can choose the evaluation methods which are right for specific needs.

**Table 5:** The list of evaluation methods in all references

Evaluation methods	Literature
ROC	[16, 21, 22, 26, 33, 35, 37, 42, 43, 47, 49]
AUC	[10, 16, 29, 33, 37, 41, 42, 49]
Univariate analysis or multivariate analysis	[6, 13, 21, 35, 46–48]
Multivariate logistic regression analysis	[8, 16, 17, 47, 48]
Linear regression analysis	[7, 18, 27]
Binary logistic regression analysis	[9, 43]
Volumetry	[44, 45]
Cox proportional hazards and logistic regression models	[46, 47]
Means of generalized estimating equations analysis	[11]
One-way analysis of variance	[22]
Correlation analysis	[22]
Bland–Altman analysis	[26]
Free-response ROC	[42]
Poisson regression	[48]
Quantitative comparison measures	[49]

In the literature cited above, the complete table about nodular signs, data analysis methods, prediction models, system evaluation and other information are shown in Table 6. As seen form the table, some studies can make horizontal comparison. For example, [16] and [37] all use same nodular signs (e.g., nodule size or lesion diameter, lobulation and spiculation), data analysis methods (e.g., Student’s t test), prediction models (e.g., PanCan model), system evaluation (AUC, ROC), data sets (e.g., DLCST database), and nodule style (e.g., pGGO, mGGO, and solid nodules). It is easy to further develop and improve in the GGN diagnosis. But most studies have different main emphasis, data sets, or nodule styles. Therefore, it’s difficult to systematically evaluate the accuracy, robustness and generalization of these studies. In addition, Table 6 indicates these models and methods considered the diagnosis of GGNs, but not aimed at them. As has been said above, GGNs are more likely to be malignant than solid nodules. The models or methods aiming at GGNs are more targeted and accurate. How to develop the models and methods aiming at GGNs is an outstanding issue. In short, further studies will be needed to develop the GGN diagnosis and formulate a unified reference standard to assess their performance.

**Table 6:** The complete table about nodular signs, data analysis methods, prediction models, system evaluation and other information

Section in this paper	Nodular signs	Data analysis methods	Prediction models	System evaluation	Data sets	Nodule style	Literature
Nodular signs	*Nodule size*	Student’s t test, Chi square test or Fisher’s exact test		Univariate and multivariate analyses	Private	GGNs	[6]
	*Nodule size, smoking history*	Student’s t test, Chi square test		Simple linear regression analysis	Private	GGNs	[7]
	*Nodule size*	Chi square test		Logistic regression method	Private	Solid nodules, mGGO, pGGO	[8]
	*Nodule size, smoking history, lung cancer history*				Private	Solid nodules, mGGO, pGGO	[12]
	*Nodule size, lobulation and spiculation, vascular convergence sign, lung cancer history, smoking history*				Private	mGGO, pGGO	[14]
	*Nodule size, lobulation and spiculation, solid components proportion, smoking history, lung cancer history*	Student’s t test, Chi square test			Private	pGGO	[15]
	*Nodule size, lung cancer history, smoking history, lobulation and spiculation, pleural indentation*	Student’s t test, Fisher’s exact test, Chi squared test		Logistic regression models	Private	Solid nodules, mGGO, pGGO	[17]
	*Nodule size, solid components proportion, lobulation and spiculation, air bronchogram*	Chi square test, Fisher’s exact test		Linear regression model	Private	GGNs	[18]
	*Lobulation and spiculation, vascular convergence sign, solid components proportion, pleural indentation*	Student’s t test, Chi square test			Private	mGGO, pGGO	[19]
	*Pleural indentation*	Chi square test			Private	mGGO, pGGO	[20]
	*Nodule size, lobulation and spiculation, air bronchogram, smoking history*	Chi square test or Fisher’s exact test, Student’s t test		Multivariate analysis, ROC	Private	pGGO	[21]
Data analysis methods	Nodule size	*Volume doubling time, mass doubling time*		Linear regression analysis	Private	GGNs	[27]
	Nodule size	*Mass doubling time, Student’s t test*			Public: NELSON	GGNs	[28]
	Nodule size	*Diffusion kurtosis imaging*		AUC, ROC	Private	Solid nodules, GGNs	[29]
	Lobulation and spiculation	*Differential geometry-based*		Percent change and proportional change	Public: LIDC-IDRI	Pulmonary nodules	[30]
	Nodule size, lobulation and spiculation	*Support vector machine*			Public: LIDC-IDRI	Pulmonary nodules	[31]
	Lobulation and spiculation	*Weighted rule based*		Leave-one-out procedure	Public: LIDC-IDRI	Pulmonary nodules	[32]
Prediction models	Nodule size, smoking history, lobulation and spiculation	Student’s t test	*Mayo clinic model*	AUC, ROC	Private	Pulmonary nodules	[33]
	Nodule sizer, lobulation and spiculation, solid components proportion, smoking history		*Modified model*	ROC, univariate analysis, multivariate analysis, stepwise logistic regression, multivariable logistic regression	Private	Solid nodules, GGNs	[35]
	Nodule size, lobulation and spiculation, lung cancer history	Student’s t test, Chi square test	*PanCan model, Lung-RADS, NCCN guidelines*	AUC, ROC	Public: DLCST	Solid nodules, mGGO, pGGO	[37]
	Nodule size, lobulation and spiculation	Machine learning	*MC-CNN*	AUC	Public: LIDC-IDRI	Pulmonary nodules	[41]
		Deep learning, machine learning	*MTANNs, CNNs*	AUC, FROC, ROC	Public	Pulmonary nodules	[42]
System evaluation	Nodule size, lobulation and spiculation, air bronchogram, pleural indentation, vascular convergence sign			*Binary logistic regression analysis, ROC*	Private	pGGO	[43]
	Nodule size, solid components proportion, lobulation and spiculation, air bronchogram	Nodule growth rate or volume-doubling time		*Volumetry*	Public: LIDC-IDRI	Solid nodules, GGNs	[44]
	Nodule size, lobulation and spiculation, smoking history	Student’s t test, Chi square test		*Volumetry*	Private	Solid nodules, GGNs	[45]
	Nodule size, solid components proportion, smoking history, lung cancer history	Chi square test or Fisher’s exact test		*Cox proportional hazards and logistic regression models, univariate and multivariate analyses*	Private	GGNs	[46]
	Nodule size, solid components proportion			*Volumetric automated computer-assisted analytic program, Cox proportional hazards and logistic regression models, multiple logistic regression analysis, univariate analysis, multiple analyses, ROC*	Private	Solid nodules, mGGO, pGGO	[47]
	Nodule size, lobulation and spiculation, smoking history			*Poisson regression, descriptive analysis, multivariable logistic regression models, multivariate analysis*	Private	Solid nodules, mGGO, pGGO	[48]
	Lobulation and spiculation	Support vector machine, hypothesis t test		*Quantitative comparison measures, AUC, ROC*	Public: LIDC-IDRI	Pulmonary nodules	[49]
Nodular signs, data analysis methods	*Nodule size, lobulation and spiculation, pleural indentation, smoking history*	*Nomogram*		Univariate analysis, multivariate analysis	Private	pGGO	[13]
Nodular signs, Prediction models	*Nodule size, lung cancer history, lobulation and spiculation*	Student’s t test, Fisher’s exact test	*PanCan model*	ROC, AUC, multivariable logistic regression analyses	Public: DLCST	Solid nodules, mGGO, pGGO	[16]
Nodular signs, system evaluation	*Nodule size, solid components proportion, vascular convergence sign, lobulation and spiculation*	Student’s t test, Chi square test		*Binary logistic regression analysis*	Private	GGNs	[9]
	*Nodule size, lobulation and spiculation*	Liner classifier		*AUC, ROC*	Private	Pulmonary nodules	[10]
	*Lobulation and spiculation*	Chi square test		*Generalized estimating equation analyses, multivariate analysis*	Private	Pulmonary nodules	[11]
	*Nodule size, lobulation and spiculation, air bronchogram*	Chi square test, Fisher exact test		*One-way analysis of variance, ROC, correlation analysis*	Private	pGGO	[22]
Data analysis methods, system evaluation	Nodule size, lobulation and spiculation	*Kurtosis and skewness of density histogram*		*ROC, Bland–Altman analysis*	Private	Solid nodules, mGGO	[26]

Italics text means main emphasis of the corresponding literature

## Conclusions

In summary, in the study of benign and malignant diagnosis for pulmonary ground glass nodules, some basic factors or characteristics are more commonly used, such as nodule size, lobulation, spiculation, the proportion of solid components within the lesion, lung cancer history, pleural indentation and so on in nodular signs, density histogram, volume doubling time and mass doubling time in data analysis methods, and the Mayo, the PanCan and other prediction models. Based on these common features, with the combination of other targeted factors, the lung CAD system can effectively improve the detection efficiency of benign and malignant identification in ground glass nodules. However, the current prediction models and data analysis methods for ground glass nodules are still limited, and the existing prediction methods still have great potential for development. In addition, with the increasing importance of pulmonary ground glass nodules diagnosis in the early diagnosis of lung cancer, methods such as deep learning and other artificial intelligence methods also significantly improve the performance of the Lung CAD system. This paper lists the relevant studies in recent years, it’s expected that with this paper the technical and clinical researchers could work out new prediction methods with better accuracy and better convenience.

## Abbreviations

GGNs: ground glass nodules
GGO: ground glass opacity
CT: computed tomography
SSN: subsolid nodule
mGGO: mixed ground glass opacity
pGGO: pure ground glass opacity
CAD: computer-aided detection
IPAs: invasive pulmonary adenocarcinomas
m-CT: mean computed tomography
DLCST: Danish Lung Cancer Screening Trial
SPNs: solitary pulmonary nodules
fGGO: the focal ground glass opacity
PET: positron emission tomography
VDT: volume doubling time
MDT: mass doubling time
DKI: diffuse kurtosis imaging
MK: mean peak
SVM: support vector machines
LU-RADS: Lung-Reporting and Data System
ACCP: American College of Chest Physicians
POM: probability of malignancy
IASLC: The International Association for the Study of Lung Cancer
ATS: American Thoracic Society
ERS: European Respiratory Society
MTANNs: massive-training artificial neural networks
CNNs: convolutional neural networks
MC-CNN: Multi-crop Convolutional Neural Network
ROC: receiver operating characteristics
AUC: area under the receiver operator characteristic curve
CBIR: content-based image retrieval
NCI: National Cancer Institute
LIDC: Lung Image Database Consortium
SDAE: stacked denoising auto-encoder
